# A nucleolin-DNMT1 regulatory axis in acute myeloid leukemogenesis

**DOI:** 10.18632/oncotarget.2131

**Published:** 2014-06-26

**Authors:** Na Shen, Fei Yan, Jiuxia Pang, Lai-Chu Wu, Aref Al-Kali, Mark R. Litzow, Shujun Liu

**Affiliations:** ^1^ The Hormel Institute, University of Minnesota, Austin, MN; ^2^ Department of Molecular and Cellular Biochemistry, The Ohio State University, Columbus, OH; ^3^ Division of Hematology, Mayo Clinic, Rochester, MN

**Keywords:** Nucleolin, DNA methyltransferase, DNA methylation, Leukemia

## Abstract

Nucleolin overexpression and DNA hypermethylation have been implicated in cancer pathogenesis, but whether and how these aberrations cooperate in controlling leukemia cell fate remain elusive. Here, we provide the first mechanistic insights into the role of nucleolin in leukemogenesis through creating a DNA hypermethylation profile in leukemia cells. We found that, in leukemia patients, nucleolin levels are significantly elevated and nucleolin overexpression strongly associates with DNMT upregulation and shorter survival. Enforced nucleolin expression augmented leukemia cell proliferation, whereas nucleolin dysfunction by RNA interference and inhibitory molecule AS1411 blocked leukemia cell clonogenic potential *in vitro* and impaired tumorigenesis *in vivo*. Mechanistic investigations showed that nucleolin directly activates NFκB signaling, and NFκB activates its downstream effector, DNA methylation machinery. Indeed, nucleolin overexpression increased NFκB phosphorylation and upregulated DNMT1 that is followed by DNA hypermethylation; by contrast, nucleolin dysfunction dephosphorylated NFκB and abrogated DNMT1 expression, which resulted in decreased global DNA methylation, restored *p15*^INK4B^ expression and DNA hypomethylation on *p15*^INK4B^ promoter. Notably, NFκB inactivation diminished, whereas NFκB overexpression enhanced *DNMT1* promoter activity and endogenous DNMT1 expression. Collectively, our studies identify nucleolin as an unconventional epigenetic regulator in leukemia cells and demonstrate nucleolin-NFκB-DNMT1 axis as a new molecular pathway underlying AML leukemogenesis.

## INTRODUCTION

Leukemia continues to be a major cause of morbidity and mortality with approximately 350,000 people diagnosed annually worldwide, and more than 250,000 deaths each year reported (www.asuragen.com). The molecular pathogenesis of this disorder is complex with constitutive activation of kinase signaling (i.e., KIT) [1], frequent gene mutations (i.e., *IDH, DNMT3A, TET2, JAK2, EZH2*) [2-4], chromosomal abnormalities (i.e., AML1/ETO, BCR/ABL) [5], overproduction of oncogenes [i.e., DNA methyltransferases (*DNMTs*), *KIT, FLT3*] [1, 6-8]] and/or aberrant epigenetics (i.e., DNA methylation, histone modifications) [5, 9]. Among them, abnormal DNA methylation, particularly at the CpG islands in the promoters of tumor suppressor genes, could be an essential player in leukemogenesis, since it strongly associates with disease progression, drug resistance and shortened survival [10, 11]. However, the molecular rules underlying DNA hypermethylation in leukemia cells are less well understood. Notably, DNA methylation involves a covalent chemical modification of DNA at the 5 position of cytosine, which is catalyzed by DNMTs, including DNMT1, DNMT3A and DNMT3B. Emerging data point out that DNMT expression is highly elevated in leukemia [6]. DNMT overexpression causes genomic DNA hypermethylation, whereas DNMT knockdown results in a reduction of DNA methylation, arguing for the crucial contribution of DNMT deregulation to the aberrant DNA methylation pattern in leukemia cells. Thus, hyperactive DNMTs have been used for the design of targeted therapy and their inhibitors (i.e., decitabine) have been in clinical use for years. However, the continuing dismal patient outcomes associated with leukemia treatment from epigenetic therapies motivated us to further investigate the molecular bases controlling *DNMT* transcription.

Nucleolin (NCL), an abundant multifunctional phosphoprotein, binds to DNA, RNA and protein, and is a highly mobile protein. On the plasma membrane, NCL serves as a binding protein for a variety of ligands implicated in cancer pathogenesis, representing a potential strategic target for effective and non-toxic cancer therapy [12-15]. In the cytoplasm, NCL functions as a mRNA stabilizer [i.e., *BCL-2* [16], *CD154* [17], *IL-2* [18] and many other tumor-related mRNAs [19]] and participates in microRNA biogenesis (i.e., *miR15*, *miR16*) [20]. In the nucleolus, NCL acts as a transcriptional factor, by directly binding to target gene promoters [21-23] or chaperoning with histone [24] to control gene expression, arguing for the complexity and the significance of NCL functions in cancer. Although NCL has been shown to be overexpressed in human malignancies [25, 26], including leukemia [14, 16], whether and how NCL upregulation promote leukemogenesis remain largely unexplored.

Previous studies suggested that NCL is likely to be involved in NFκB signaling [27], as NCL inhibitor AS1411 impairs the function of IkappaB kinase, an NFκB activator. We also found that Sp1 physically interacts with NFκB to transactivate *DNMT1* through promoter binding [7]. However, the regulatory roles of NCL in NFκB activity or NFκB itself in *DNMT1* gene transcription need to be precisely defined. Given that the aberration of both NCL and DNMTs frequently occurs in cancers [16, 25, 28], we hypothesized that NCL may accelerate leukemogenesis via DNMT1-dependent DNA hypermethylation that is driven by NCL-NFκB cascade. To test this hypothesis, we examined the contribution of NCL to leukemia cell proliferation and tumor growth. We delineated the mechanistic pathways controlling NCL-augmented leukemogenesis and validated NCL as a novel DNA methylation regulator. We demonstrated that NCL upregulates DNMT1 gene expression via NFκB signaling. Genetic deletion or pharmacological inhibition of NCL induced global and gene specific DNA hypomethylation and the subsequent leukemia regression. Thus, we proposed a model in which NCL overproduction enhances NFκB activity, leading to aberrant DNA methylation that confers leukemia cells with a strong growth advantage.

## RESULTS

### NCL is upregulated in leukemia patients and NCL levels are positively correlated with leukemia cell proliferation

**Figure 1:**
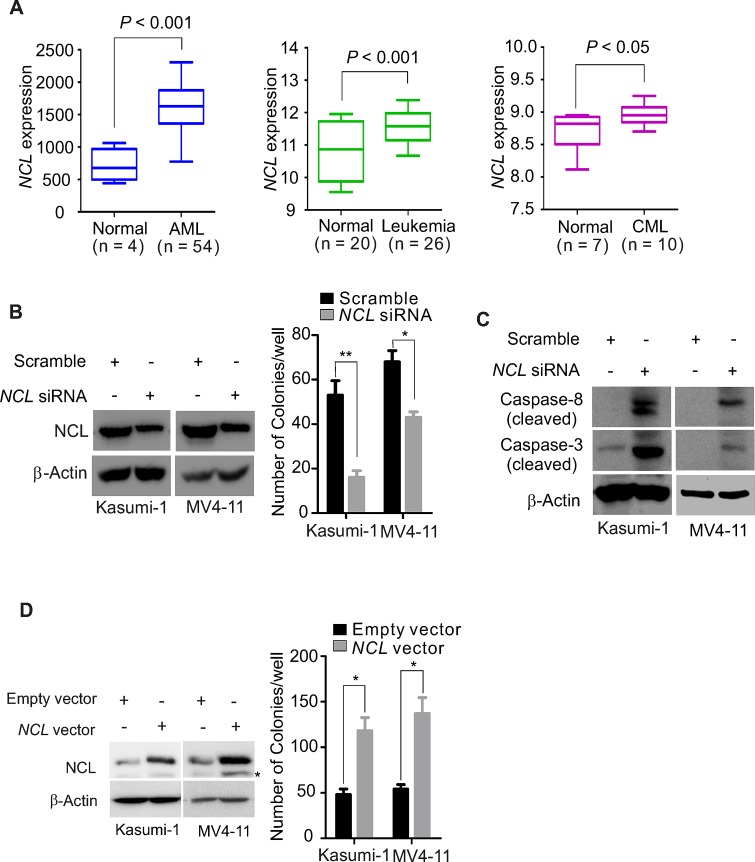
NCL deregulation significantly influences leukemia cell growth A, GEO data were analyzed for *NCL* expression in leukemia patients versus normal control. Bars represent mean and range of each group. B, Kasumi-1 or MV4-11 cells were transfected with *NCL* siRNA or scramble. Left: NCL expression measured by Western blot at 48 hour after transfection; Right: at 6 hour after transfection, about 500 cells were applied to colony-forming assays. Graph is the quantification of colony number from three independent experiments, mean ± SD, *, *P* < 0.05; **, *P* < 0.01. C, Kasumi-1 or MV4-11 cells were transfected with *NCL* siRNA or scramble for 48 hours and the cells were lysed for Western blot using indicated antibodies. D, Kasumi-1 or MV4-11 cells were transfected with *NCL* expression or empty vector. Left: *NCL* expression determined by Western blot at 48 hour after transfection, * indicates the non-specific band; Right: at 6 hour after transfection, about 500 cells were subjected to colony-forming assays. Graph is the quantification of colony number from three independent experiments, mean ± SD, *, *P* < 0.05.

To determine NCL expression in leukemia, we obtained gene microarray data from three GEO (Gene Expression Omnibus) databases, GPL201 (CML, n = 10; normal, n = 7) (GSE5550) [29], GPL8300 (AML, n = 54; normal, n = 4) (GSE2191) [30], GPL96 (AML, n = 26; normal, n = 20) (GSE9476) [31], in which the gene expression was assessed using Affymetrix U133Plus2.0 GeneChips and analyzed by GraphPad Prism 5.04. By such global transcriptional profiling analysis, we found that *NCL* levels are highly elevated in leukemia patients as compared to the corresponding normal controls in all three datasets (Fig. 1A).

To address whether NCL deregulation contributes to leukemia cell proliferation, the “loss- and gain-of-function” approach was employed to examine how leukemia cells respond to the change in NCL levels. Initially, we knocked down the *NCL* gene expression in AML cell Kasumi-1 or MV4-11 by siRNA. Scrambled-transfected cells were used as negative control. Because NCL is a phosphorylated protein [32] and the phosphorylation status critically impacts its activity and cellular localization [33], we first examined NCL phosphorylation status. As shown in Supplementary Fig. S1, specific depletion of *NCL* abolished its phosphorylation, suggesting the potential application of protein downregulation in blocking NCL activity. To prove the biological significance of *NCL* gene depletion, we conducted colony-forming assays with the aforementioned transfected cells. As shown in Fig. 1B, *NCL* knockdown led to a significant decrease of the colony number in both Kasumi-1 (51.25 ± 8.09 versus 16.5 ± 2.65; *P* < 0.01) and MV4-11 (64.25 ± 5.56 versus 40.25 ± 2.5; *P* < 0.05). It is reported that caspase-8 is an initiator caspase, and once activated, it activates effector caspases (i.e., caspase-3) [34]. Further, caspase-3 is an essential ‘executioner’ caspase, and the high levels of inactive caspase-3 associates with unfavorable prognosis in leukemia [35]. Therefore, we measured the status of caspase-8 and caspase-3, and found that siRNA-mediated NCL depletion dramatically increases the expression of cleaved form of both caspase-8 and caspase-3 (Fig. 1C), indicating that targeted NCL depletion activates the caspase pathway.

To further characterize the role of NCL overproduction in leukemia growth, Kasumi-1 or MV4-11 cells were transfected with *NCL* expression or empty vector and subjected to colony-forming assays at 48 hours after transfection. The enforced *NCL* expression at both RNA and protein levels was confirmed by Western blot or PCR (Fig. 1D, left). We found that the cells carrying *NCL* overexpression have markedly higher colony number in Kasumi-1 (118.2 ± 14.6 versus 48 ± 6.3; *P* < 0.05) and MV4-11 (137.4 ± 17.5 versus 54 ± 5.1; *P* < 0.05), when compared to the empty vector group (Fig. 1D, right). Together, these results indicate that NCL deregulation critically contributes to leukemia cell expansion.

### *NCL* knockdown impairs leukemia cell oncogenic potential *in vivo*

**Figure 2:**
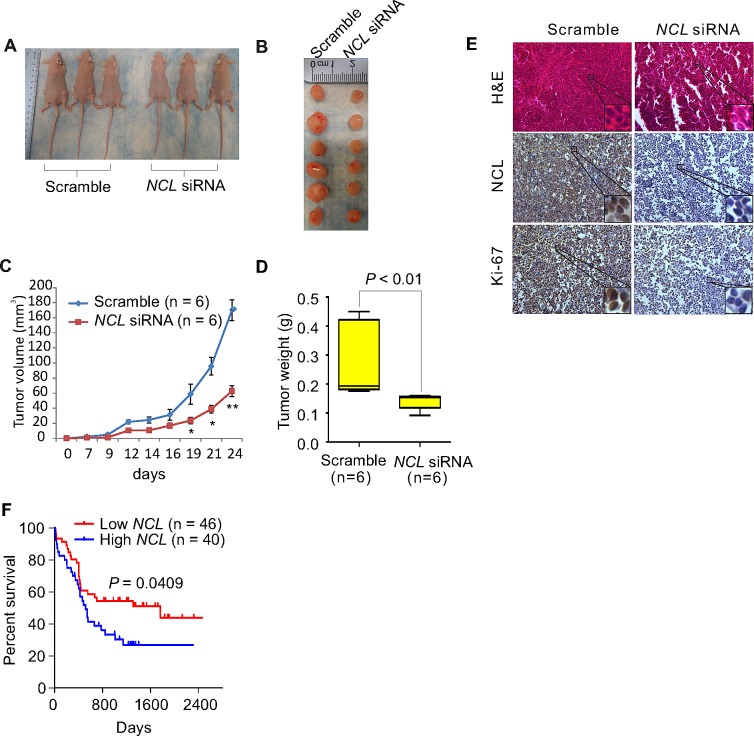
NCL aberrations contribute to the survival and expansion of the leukemia cells *in vivo* A, Pictured are the external views of tumor-bearing nude mice. MV4-11 cells were transfected with *NCL* siRNA or scramble, 2 × 10^6^ cells were resuspended in 50% BD Matrigel ^™^ matrix and injected subcutaneously into both flanks of 4–6 weeks old mice (n = 3 mice/group). B, Pictured are the tumors derived from tumor-bearing nude mice reported in (A) (n = 6 tumors/group). C, The tumor growth curve compares the growth rate of tumors between siRNA and scramble groups. Bars represent mean ± SEM, *, *P* < 0.05; **, *P* < 0.01. D, The graph indicates the tumor weight derived from (A) (n = 6 tumors/group). Bars represent mean ± SEM, *P* < 0.01. E, H&E or IHC staining of tumor sections derived from (A) using indicated antibodies (Original magnification × 200). F, The Kaplan-Meier curve (5-year survival) comparing low and high *NCL* expression in leukemia patients with normal karyotype from GEO dataset (GSE16432, GPL10105–10108, n = 86). The log-rank was used to evaluate the differences among survival distribution. Patients were grouped into quartiles according to *NCL* expression levels (each quartile containing ~50% of patients) and divided into high *NCL* and low *NCL*.

The demonstration of NCL deregulation in controlling leukemia cell growth *in vitro* motivated us to further examine its role *in vivo*. Thus, MV4-11 cells were transfected with *NCL* siRNA or scramble, and at 6 hour after transfection when the cells did not undergo apoptosis, 2 × 10^6^ of transfected cells were mixed with 50% BD Matrigel^™^ matrix and subcutaneously injected into both flanks of 4–6 week old nude mice (n = 3 mice/group). Notably, the effect of small synthetic oligos (i.e., siRNA) on tumorigenesis has been well-documented [36]. We found that at 24 days post injection, mice inoculated with *NCL* knockdown cells have a considerable reduction of tumor volume compared to mice receiving scramble control cells (63.1% decrease, *P* < 0.01) (Fig. 2A and B). In addition, cells transfected with scramble formed tumors (incidence: 100%) in 4 to 6 days, whereas tumors from siRNA-transfected cells were palpable (incidence: 100%) only after 8 to 10 days post injection (Fig. 2C). Tumors from siRNA-transfected cells weighed 52.1% (*P* < 0.01) less than those derived from scramble-transfected cells (Fig. 2D). Moreover, as shown in Fig. 2E and supplementary Fig. S2, H&E staining revealed that the tumor formation is significantly suppressed, and immunohistochemistry (IHC) staining with Ki-67, a cellular marker of proliferation, illustrated that tumor cell proliferation is remarkably attenuated in the *NCL* knockdown group. Of note, IHC staining with NCL antibody indicated the reduced NCL expression in tumors from siRNA-transfected cells when compared to the scramble. Such tumor regression induced by *NCL* depletion inspired us to address whether NCL levels associate with patient survival. To this end, we obtained GEO dataset GSE16432 [GPL10105–10108, myeloid leukemia [37]], which includes gene expression profiling and clinical outcome data of 86 patients. For statistical analysis, these patients were divided into high or low *NCL* expression. In line with our discoveries showing the positive correlation between NCL levels and leukemia cell proliferation rate *in vitro* and in mice, leukemia patients with low *NCL* expression survived longer than those with high *NCL* expression (*P* = 0.0409) (Fig. 2F). Thus, limiting NCL expression in leukemia cells restrains leukemogenesis and represents a distinctive approach in leukemia therapy.

### NCL positively regulates *DNMT* gene expression

**Figure 3:**
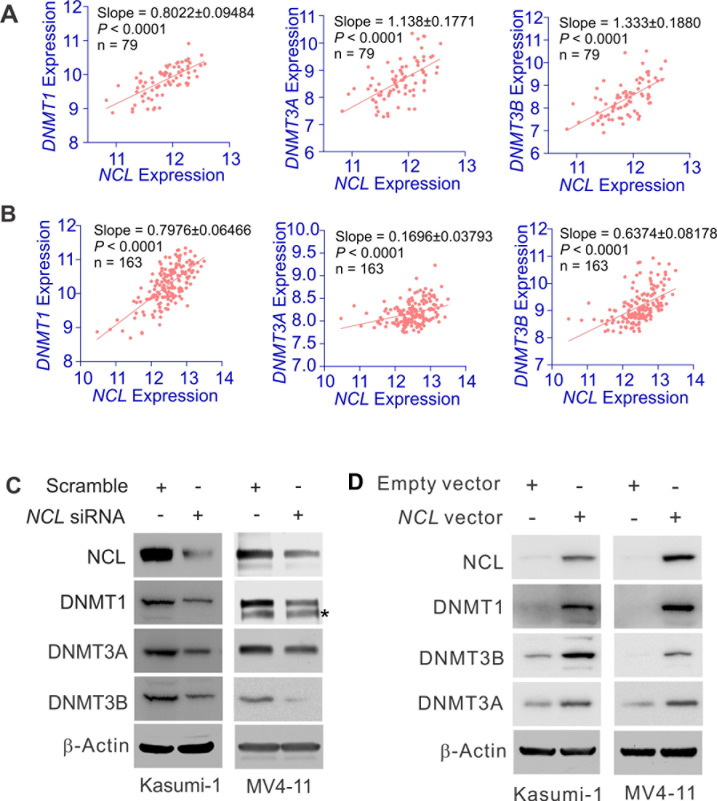
NCL positively regulates DNMT expression A and B, The analysis of GEO dataset GSE12417 [GPL570, AML, n = 79 (A); GPL96, AML, n = 163 (B)] showing the correlation between *NCL* and *DNMT* expression in leukemia patients. Correlation between *NCL* and *DNMT1* or *DNMT3A* or *DNMT3B* was assessed by Spearman correlation. *P* < 0.05 was considered statistically significant. C, MV4-11 or Kasumi-1 cells were transfected with *NCL* siRNA or scramble for 48 hours and the expression of targeted genes were detected by Western blot. * indicates the non-specific band. D, Kasumi-1 or MV4-11 cells were transfected with *NCL* expression or empty vector for 48 hours and the cells were lysed for Western blot.

Given the higher levels of both NCL and DNMTs in leukemia, NCL overexpression may augment leukemogenesis through aberrant DNMT expression. To test this hypothesis, initially we obtained GEO dataset GSE12417 [GPL570, AML, n = 79; GPL96, AML, n = 163 [38]], regarding *NCL* and *DNMT* expression in leukemia patients. We analyzed this dataset by Spearman correlation, which measures the correlation of rank patient ordering between two values (i.e., *NCL* or *DNMT*) at *P* < 0.05 stringency. Interestingly, we observed that in leukemia patients, higher levels of *DNMT* are accompanied by *NCL* overexpression, while lower expression of *DNMT* is seen in the patients carrying lower NCL transcription, indicative of a positive correlation between NCL and *DNMT* transcripts (Fig. 3A-B), which was independently confirmed by other datasets, GSE16432 (myeloid leukemia, GPL10105–10108, n = 86; GPL8651–8653, n = 88) (Supplementary Fig. S3). Thus, *NCL* and *DNMT* oncogenic pathways may exert a regulatory interaction in leukemia cells.

To address whether *NCL* modulates DNMT expression, targeted-depletion of *NCL* expression by siRNA was performed in Kasumi-1 and MV4-11 cells. The transfected cells were collected at 48 hours and the expression of *NCL* and DNMT was measured by Western blot and qPCR. As shown in Fig. 3C and Supplementary Fig. S4 (left), *NCL* knockdown by siRNA suppressed the expression of DNMT1, DNMT3A and DNMT3B at both mRNA and protein levels. To further clarify the regulatory role of *NCL* in *DNMT* expression, we overexpressed *NCL* in Kasumi-1 or MV4-11 cells and found that *NCL* overexpression leads to the upregulation of DNMT1, DNMT3A and DNMT3B at both mRNA and protein levels (Fig. 3D and Supplementary Fig. S4, right). Although previous investigations showed that *NCL* functions as an mRNA stabilizer [16-18], our observation, which the change of *NCL* is positively correlated with the change of *DNMT* mRNA levels, also raise another possibility that *NCL* acts as a regulator of *DNMT* gene transcription.

### NCL-mediated *DNMT1* deregulation occurs through NFκB signaling

**Figure 4:**
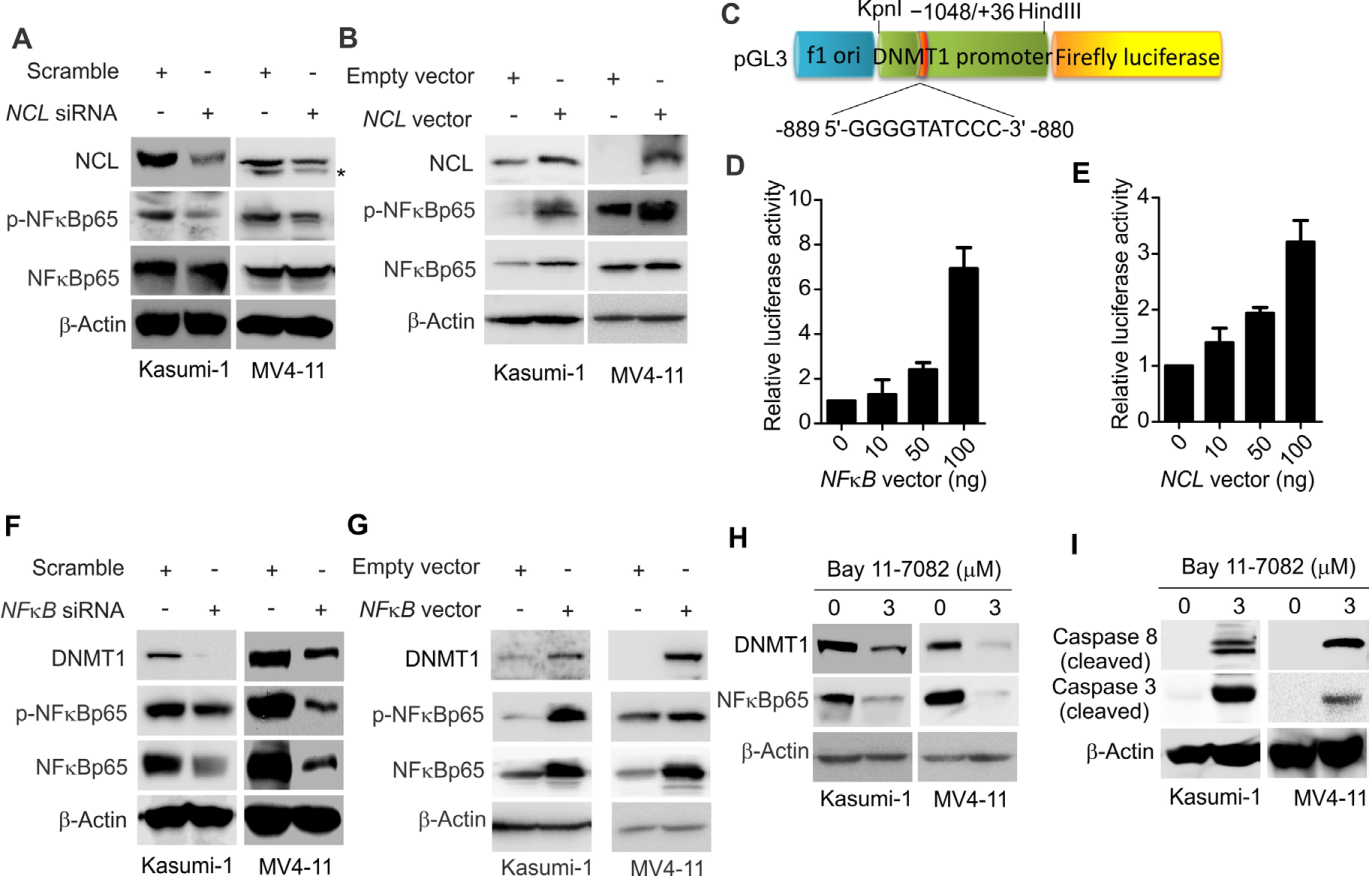
NCL modulates DNMT1 expression via NFκB pathway A, Kasumi-1 or MV4-11 cells were transfected with *NCL* siRNA or scramble for 48 hours and the cells were lysed for Western blot. * indicates the non-specific band. B, Kasumi-1 or MV4-11 cells were transfected with *NCL* expression or empty vector for 48 hours and subjected to Western blot. C, Schematic representation of luciferase constructs used for reporter assays. D, pGL3-*DNMT1* constructs were co-transfected with *NFκB* expression or empty vector into 293T cells and luciferase activity was measured at 48 hours after transfection. E, 293T cells were co-transfected with pGL3-*DNMT1* and *NCL* expression or empty vector. The luciferase activity was measured at 48 hour after transfection. F, MV4-11 or Kasumi-1 cells were transfected with *NFκB* siRNA or scramble for 48 hours and subjected to Western blot. G, Western blot to determine the alteration of NFκB targets in MV4-11 or Kasumi-1 cells transfected with *NFκB* expression or empty vector for 48 hours. H, Western blot for indicated target genes in MV4-11 or Kasumi-1 cells treated with NFκB inhibitor Bay 11-7082 for 12 hours. I, Western blot for the cleaved caspases in MV4-11 or Kasumi-1 cells exposed to Bay 11-7082 for 12 hours.

AS1411, an antiproliferative G-rich phosphodiester oligonucleotide, is an inhibitor for NCL function [27]. The observed inhibitory effect of AS1411 on phosphorylation of IKK complex [27], a central regulator of NFκB activity, indicated that NCL is likely to be involved in NFκB signaling. To test this, Kasumi-1 and MV4-11 cells were transfected with *NCL* siRNA, and at 48 hour after transfection, the cell lysates were subjected to Western blot. As shown in Fig. 4A, the phosphorylation of NFκBp65 (ser536), an indicator of NFκB activation, was remarkably decreased in the presence of *NCL* siRNA. When Kasumi-1 and MV4-11 cells were transfected with *NCL* expression or empty vector, we evidenced that ectopic *NCL* expression increases NFκB phosphorylation (Fig. 4B), suggesting that NCL deregulation is a contributor to aberrant NFκB signaling in leukemia cells.

By using bioinformatic analysis, we found one putative NFκB binding site (GGGGTATCCC) in 5'-flanking region 1 kb upstream of the transcription start site (TSS) of *DNMT1* promoter. We then cloned *DNMT1* promoter region surrounding the NFκB binding element into luciferase reporter (pGL3-*DNMT1*) (Fig. 4C), and cotransfected pGL3-*DNMT1* with NFκB expression (10, 50, 100 ng) or empty plasmid in 293T cells, reporter assays showed that the luciferase activity driven by *DNMT1* promoter is enhanced by *NFκB* overexpression in a dose-dependent fashion (2.05, 2.38 or 7.52 folds, Fig. 4D). Since NFκB activity is under control of NCL, we cotransfected pGL3-*DNMT1* with *NCL* expression (10, 50, 100 ng) or empty plasmid in 293T cells for 48 hours. As expected, *DNMT1* promoter luciferase activity was increased up to 1.23, 1.92 or 3.64 folds (Fig. 4E). To further document the role of NFκB in DNMT1 regulation, we used siRNA to specifically knock down NFκB in Kasumi-1 and MV4-11 cells, and found that endogenous DNMT1 expression is remarkably downregulated at both RNA and protein levels (Fig. 4F and supplementary Fig. S5, left). By contrast, when *NFκB* was overexpressed in Kasumi-1 or MV4-11 cells, *DNMT1* was significantly upregulated (Fig. 4G and Supplementary Fig. S5, right). Notably, the expression of DNMT3A and DNMT3B was also changed in response to the alteration of NFκB activities (not shown). Finally, when Kasumi-1 or MV4-11 cells were treated with 3 M of Bay 11-7082, a specific NFκB inhibitor [1], for 12 hours, DNMT1 expression was considerably reduced (Fig. 4H), and the cleaved form of caspase-3 and caspase-8 was markedly increased (Fig. 4I). Together, these findings indicate that DNMT1 expression in leukemia cells is controlled, at least partially, by a NCL-NFκB axis.

### NCL deregulation alters DNA methylation profile

**Figure 5:**
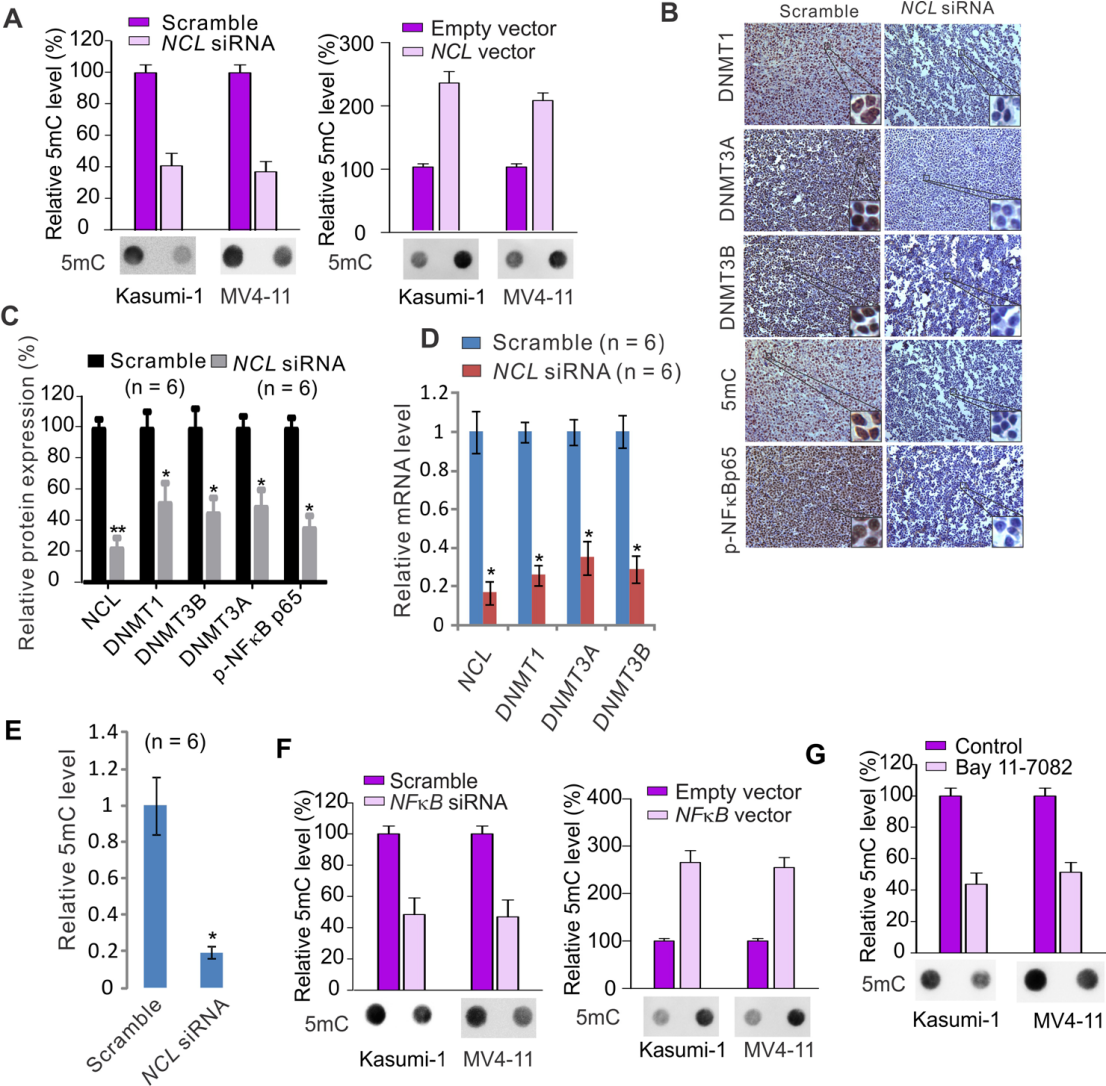
NCL dysfunction modulates DNA methylation program A, MV4-11 or Kasumi-1 cells transfected with *NCL* siRNA or scramble (left) or *NCL* expression or empty vector (right) for 48 hours, the genomic DNA was extracted and subjected to Dotblot using 5mC antibody. B, IHC staining of tumor sections reported in Figure 2B using antibodies specific for DNMTs, phospho-NFκBp65 and 5mC (Original magnification × 200). C, The cell lysates from individual tumors reported in Figure 2B (n = 6) were subjected to Western blot using indicated antibodies. Graph is the quantification of target protein bands, which were normalized by the corresponding β-actin, and represents mean ± SD. *, *P* < 0.05, **, *P* < 0.01. D, qPCR showing the change of NCL and DNMTs in tumors reported in Figure 2B (n = 6). *, *P* < 0.01. E, Dotblot for the alteration of global DNA methylation in tumors reported in Figure 2B (n = 6). Graph is the quantification of Dotblot intensity from individual tumor and represents mean ± SD. *, *P* < 0.05. F, Kasumi-1 or MV4-11 cells were transfected with *NFκB* siRNA or scramble (left) or *NFκB* expression or empty vector (right) for 48 hours, the genomic DNA was extracted and subjected to dotblot using 5mC antibody. G, Genomic DNA was extracted from MV4-11 or Kasumi-1 cells treated with Bay 11-7082 for 12 hours and subjected to Dotblot using 5mC antibody. Regarding the Dotblot in cell lines, lower: representative image of Dotblot; upper: the quantification of dot intensities from three independent experiments, and graphs represent mean ± SD.

Given the positive regulatory role of NCL in *DNMT* gene transcription, next we asked whether NCL plays a role in reforming the DNA methylation profile. Kasumi-1 or MV4-11 cells were transfected with *NCL* siRNA for 48 hours, genomic DNA was prepared using DNA Extraction Kit (Qiagen) and subjected to Dotblot. The quantitative traits of Dotblot method for genomic DNA methylation were validated by using the serial dilution of standard unmethylated (C) or methylated (5mC) to test the sensitivity and specificity of 5mC antibody, and using the methylene blue staining to determine the loading equality (Supplementary Fig. S6). The dynamic range of detection of 5mC implied that these assays are reliable to determine differences in the 5mC content. As shown in Fig. 5A (left), the global DNA methylation was greatly decreased after *NCL* targeted-depletion. In contrast, when *NCL* was overexpressed in Kasumi-1 or MV4-11 cells, the DNA methylation was significantly increased (Fig. 5A, right). To recapitulate the NCL-mediated change of DNA methylation program *in vivo*, we employed IHC staining to assess the alterations of NCL targets in tumors described in Fig. 2B using antibodies against DNMTs, 5mC or phospho-NFκB. As shown in Fig. 5B and Supplementary Fig. S7, IHC staining showed that DNMT downregulation and NFκB dephosphorylation occur with a concurrent reduction of global DNA methylation in tumors from *NCL*-knockdown cells when compared to scramble group, which was further verified by the molecular characterization of same tumors using Western blot, PCR and Dotblot (Fig. 5C-E).

Having demonstrated that NFκB functions as a downstream mediator of NCL pathway and a positive regulator of *DNMT1* gene expression, next we sought to determine how DNA methylation is changed in response to NFκB modulation. Similar to the alteration of NCL activity, Dotblot showed that NFκB downregulation by siRNA leads to a reduction of global DNA methylation (Fig. 5F, left), while NFκB overexpression results in a significant increase of global DNA methylation in both Kasumi-1 and MV4-11 cells (Fig. 5F, right). Moreover, when Kasumi-1 or MV4-11 cells were exposed to Bay 11-7082 (3 μM) for 12 hours, the Dotblot identified a remarkable decrease of global DNA methylation in the presence versus absence of Bay 11-7082 (Fig. 5G). Altogether, these findings suggest that NCL and NFκB aberrations control, at least partially, the abnormal DNA methylation program in leukemia cells.

### Exposure to NCL inhibitor AS1411 leads to DNA hypomethylation and the blockage of leukemia cell growth

**Figure 6:**
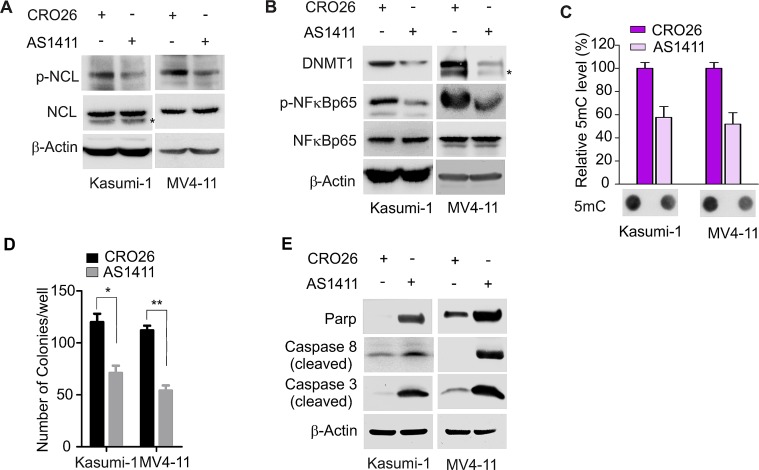
Pharmacological inhibition of NCL reduces DNA methylation and suppresses leukemia cell growth A, MV4-11 or Kasumi-1 cells were treated with AS1411 or CRO26 (1 or 3 μM, respectively) for 48 hours and the cells were lysed for Western blot. p-NCL, phosphorylated NCL. * indicates the non-specific band. B, Western blot to detect the change of NFκB and DNMT1 in MV4-11 or Kasumi-1 cells treated with AS1411 or CRO26 (1 or 3 μM, respectively) for 48 hours. p-NFκB, phosphorylated NFκB. * indicates the non-specific band. C, Genomic DNA was extracted from the above treated MV4-11 or Kasumi-1 cells and subjected to Dotblot using 5mC antibody. Lower: representative image of Dotblot; Upper: the quantification of dot intensities, mean ± SD. D, MV4-11 or Kasumi-1 cells were treated with 1 or 3 μM of AS1411 or CRO26, respectively, and subjected to colony-forming assays. The graph indicates the colony number from three independent experiments. *P* values were determined by a two-tailed Student's t-test, mean ± SD, *, *P* < 0.05; **, *P* < 0.01. E, Western blot showing the change of cleaved caspases in MV4-11 or Kasumi-1 cells treated with 1 or 3 μM of AS1411 or CRO26 for 48 hours, respectively.

Next we determined whether pharmacological modulation of NCL oncogenic pathway can restore the deregulated DNA methylation machinery. We focused on AS1411, which is an antiproliferative G-rich phosphodiester oligonucleotide, NCL-binding aptamers [27] and is in phase II clinical trials [39]. Given that NCL is a phosphorylated protein [32], initially we investigated whether AS1411 treatment impairs NCL phosphorylation. When Kasumi-1 or MV4-11 cells were exposed to AS1411 at the concentrations of 3 μM or 1 μM, respectively, for 48 hours, Western blot revealed a decrease of NCL phosphorylation with no obvious change of total protein levels (Fig. 6A), thus identifying NCL dephosphorylation as an unappreciated mechanism underlying AS1411-antileukemic activities.

Because NCL regulates NFκB-dependent DNMT1 expression, we asked whether the anticancer actions of AS1411 occur via DNA methylation inhibition. To address this, we treated Kasumi-1 or MV4-11 cells with 3 μM or 1 μM of AS1411, respectively, for 48 hours, and found that NFκB is dephosphorylated in the presence of AS1411, further supporting NCL as a upstream regulator of NFκB pathway, which was followed by the abrogation of DNMT1 expression at both RNA and protein levels (Fig. 6B and Supplementary Fig. S8). Dotblot revealed that global DNA methylation is significantly decreased upon exposure to AS1411 (Fig. 6C). The biological significance of AS1411-induced DNA hypomethylation was supported by the decrease of colony numbers (Fig. 6D) and the increase of activated caspase-3, caspase-8 and Parp in AS1411-treated cells versus CRO26 control (Fig. 6E). Together, these data demonstrate that AS1411 exerts its antileukemic activities through its DNA hypomethylating effect.

### NCL inactivation restores the expression of tumor suppressor gene *p15^INK4B^* via promoter DNA hypomethylation

**Figure 7:**
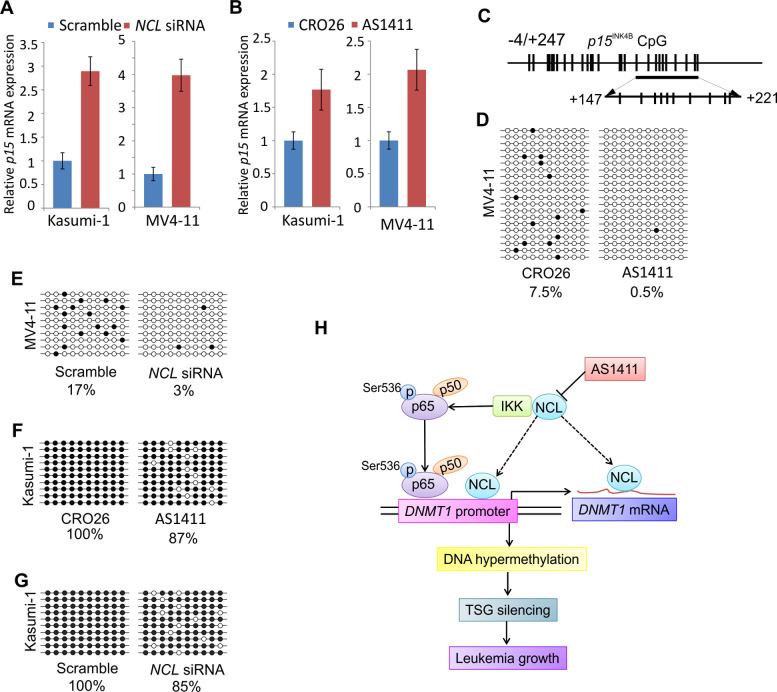
NCL inactivation contributes to the restoration of *p15*^INK4B^ gene in leukemia cells A and B, qPCR analysis of *p15*^INK4B^ expression in MV4-11 or Kasumi-1 cells transfected with *NCL* siRNA (A) or treated with AS1411 (B) as well as the corresponding controls for 48 hours. C, Schematic representation of CpG sites in *p15*^INK4B^ promoter for Bisulfite sequencing. CpG locations are indicated as vertical bars in the promoter of *p15*^INK4B^ (top). Arrows indicate the bisulfite sequencing region (bottom). D and E, Bisulfite sequencing of *p15*^INK4B^ gene promoter in MV4-11 cells treated with NCL inhibitor (D) or transfected with *NCL* siRNA (E) for 48 hours. F and G, Bisulfite sequencing of *p15*^INK4B^ gene promoter in Kasumi-1 cells treated with NCL inhibitor (F) or transfected with *NCL* siRNA (G) for 48 hours. H, A working model illustrating the role of NCL-NFκB cascade in regulation of DNMT1-dependent DNA methylation program in leukemia cells. Note, solid lines represent pathways that are identified in the present study, dash lines indicate pathways that remain elusive. D-G, Open circles indicate unmethylated CpG sites, filled circles indicate methylated CpG sites. Results of 20 or 10 clones are presented.

It is reported that the tumor suppressor gene (TSG) *p15*^INK4B^ is silenced through promoter DNA hypermethylation in leukemia cells [10]. Given that the NCL abnormality positively alters global DNA methylation program in leukemia cells, we speculated that NCL may negatively regulate the expression of *p15*^INK4B^. Because *p15*^INK4B^ is barely detectable in leukemia cells, to test our hypothesis, we mainly focused on the aspect of *p15*^INK4B^ upregulation and NCL inactivation using siRNA or AS1411 in Kasumi-1 or MV4-11 cells. As expected, qPCR demonstrated that *p15*^INK4B^ gene was significantly increased (2.89 fold in Kasumi-1 and 3.98 fold in MV4-11) in response to *NCL* siRNA, when compared to the scramble-treated cells (Fig. 7A). *NCL* siRNA-induced restoration of *p15*^INK4B^ expression was further evident by AS1411 treatment showing that *p15*^INK4B^ transcription is increased by 1.76 fold in Kasumi-1 or 2.06 fold in MV4-11, when compared to CRO26 control (Fig. 7B).

To elucidate the mechanisms underlying *p15*^INK4B^ upregulation by NCL inhibition, we examined the DNA methylation status of *p15*^INK4B^ gene promoter by bisulfite sequencing. We focused on a region in *p15*^INK4B^ gene promoter located between nucleotide −4 and +247 relative to the transcription start encompassing 27 CpG sites (Fig. 7C). When the genomic DNA was prepared, bisulfite converted, PCR amplified and cloned, the sequencing from 10 or 20 clones showed that in MV4-11, NCL inhibition by AS1411 decreased the methylation of *p15*^INK4B^ promoter from 7.5% (negative control, CRO26) to 0.5% (AS1411 treatment) (Fig. 7D), but *NCL* knockdown by siRNA reduced the methylation from 17% (scramble) to 3% (siRNA transfection) (Fig. 7E). Further, DNA methylation of *p15*^INK4B^ promoter was decreased from 100% to 87% in the presence of AS1411 (Fig. 7F) or from 100% to 85% after *NCL* siRNA transfection (Fig. 7G) in Kasumi-1 cells when compared to the negative control. In all, our results demonstrated that NCL overexpression enhances NFκB activity and subsequently promotes DNMT1 expression, which is followed by global DNA hypermethylation and epigenetic silencing of TSG transcription, thus, conferring a survival advantage to leukemia cells (Fig. 7H).

## DISCUSSION

NCL functions and DNMT activities are involved in many fundamental cellular processes and the contribution of their abnormalities to cancer pathogenesis has been documented. Owing to this, inhibitors targeting NCL or DNMT have been developed and are in clinical use for decades. However, continued poor clinical outcomes call for further investigation of whether and how these signaling pathways are regulated, and whether and how they have functional interactions. In this report, we present evidence that 1) In leukemia patients, high NCL expression predicts a poor prognosis; 2) Enforced *NCL* expression increases leukemia cell proliferation, whereas the disruption of *NCL* gene abundance inactivates NCL leading to the blockage of leukemia growth; 3) The expression of DNMT is positively correlated with the expression of NCL in leukemia patients. Further, NCL upregulates *DNMT1* gene transcription through NFκB signaling, which is followed by DNA hypermethylation *in vitro* and *in vivo*; 4) Pharmacological modulation of NCL and NFκB reduces global DNA methylation and restores TSG expression via promoter hypomethylation. These findings identify a novel molecular process underlying NCL-accelerated leukemogenesis and the anticancer actions of AS1411, uncover NCL as an unconventional DNA methylation modifier and epigenetic target for therapy, and develop AS1411 as a promising DNA hypomethylating agent as a single or in combination forms.

NCL is an abundant non-ribosomal protein shuttling among the plasma membrane, cytoplasm, nucleolus and nucleoplasm [14, 33, 40, 41]. It is a multifunctional protein capable of interacting with DNA, RNA and protein thereby governing gene expression [42], miR biogenesis [20, 43] and RNA stability [16]. The overexpression of NCL frequently occurs in cancers [25, 26] and is positively correlated with worse clinical outcomes [28], which is consistent with our discoveries that leukemia patients with higher NCL levels have shorter survival than those with lower NCL expression. The disruption of *NCL* gene expression in Kasumi-1 or MV4-11 cells suppresses leukemia cell expanding *in vitro* and leads to tumor regression *in vivo*; In contrast, NCL overexpression in these cells increases colony-formation capability. The following question is how NCL aberrations determine leukemia cell fate. While a recent report showed that NCL may impact the methylation status of *rRNA* gene promoter in Arabidopsis [44], the roles of NCL in *DNMT* gene transcription and global DNA methylation in cancer have not been described. By analyzing the GEO datasets, we found that *NCL* levels are in parallel with the expression of *DNMT1, DNMT3A* and *DNMT3B* in leukemia patients. Using “gain- and loss-of-function” strategies, we demonstrated that NCL aberrations positively modulate DNMT expression and DNA methylation program *in vitro* and *in vivo*. NCL inactivation restored *p15*^INK4B^ expression through its promoter demethylation. To this end, we believe that this is the first comprehensive study of the functional role of *NCL* in epigenetic regulation with clear clinical implications.

It has been shown that an NCL inhibitor, AS1411, regulates the phosphorylation status of IKK complex [27], a central regulator of NFκB activity, suggesting that NCL may be involved in the modulation of NFκB function. Given the likely “off-target” effects from AS1411 treatment, further investigations are required to document the contribution of NCL to NFκB activity. To this end, we employed “gain- and loss-of-function” approach to specifically manipulate NCL expression. Our results showed that *NCL* overexpression augments, whereas the inactivation of *NCL* by siRNA decreases, the phosphorylation of NFκBp65 (Ser536), thus, demonstrating NCL as an upstream regulator of NFκB signaling. Given that IKK induces the phosphorylation of NFκBp65 at the site of Ser536 [45], it is likely that NCL promotes NFκB phosphorylation through its physical interaction with IKK complex. Further, although NFκB has been shown to physically interacts with Sp1 and then transactivates *DNMT1* [7], the direct influence of NFκB itself on *DNMT1* transcription and global DNA methylation have not been clearly defined. While NFκB can be recruited by Sp1 to *DNMT1* gene promoter [7], we found here that DNMT1 promoter contains NFκB binding site. The change of NFκB positively matches the alterations of *DNMT1* promoter activity and endogenous DNMT1 expression in leukemia cells. These observations suggest that NFκB activity controls DNMT1 expression through 1) the binding of itself or/and 2) the recruitment by Sp1 to *DNMT1* promoter. It is worth noting that NCL expression also positively associates with the levels of DNMT3A and DNMT3B in leukemia patients. While the underlying mechanisms remain elusive, NFκB activity could be critical too. Indeed, our results indicate that NFκB overexpression enhanced, whereas NFκB suppression abrogated, the transcription of *DNMT3A* and *DNMT3B*. However, it is unclear how NFκB controls DNMT3A and DNMT3B expression. It may occur through NFκB-suppressed *miR-29b* [1], since *miR-29b* directly binds to the 3' untranslated region of *DNMT3A* and *DNMT3B* mRNA in leukemia [46]. Given the essential roles of DNMT3A and DNMT3B in *de novo* DNA methylation, further characterization of the molecular processes controlling NCL-associated upregulation of DNMT3A and DNMT3B will gain more insights into not only the NCL-accelerated leukemogenesis, but also the cause of DNA hypermethylation in leukemia cells. Thus, these findings have, for the first time, clearly proven that the NCL-NFκB axis governs the DNA methylation program.

Because of the importance of NCL function in cancer, an NCL inhibitor, AS1411, was designed and has been used in clinical trials. It is reported that AS1411 binds to cell surface NCL, leading to the inhibition of DNA replication [47] and the destabilization of *Bcl-2* mRNA [13]. However, the precise mechanisms of the antiproliferative effects of AS1411 are largely unclear. Recently, Pichiorri *et al* reported that AS1411 participates in miR regulation [43]. Here, we found that AS1411 treatment impairs NCL phosphorylation. Further, upon exposure to AS1411, the expression of DNMTs was downregulated, and the levels of global DNA methylation were reduced. Importantly, AS1411 treatment restored the expression of epigenetic-silenced *p15*^INK4B^ gene via its promoter hypomethylation. Collectively, not only do these discoveries identify a new molecular rule behind the anticancer actions of AS1411, but also establish the feasibility of applying AS1411 as a hypomethylating agent to treat leukemia patients unresponsive to current methylation inhibitors as single or combination agent.

Although the contribution of DNMT aberrations to human malignancies has been appreciated [6, 7, 46, 48], the molecular processes underlying such abnormalities are incompletely understood. We and others previously demonstrated that DNA hypermethylation is under the control of miRs [46], Sp1/NFκB complex [7], TET or IDH mutations [3, 49]. Here we showed that DNMT expression and DNA methylation profile are regulated by NCL-NFκB axis in leukemia cells. Given the in-parallel association between *NCL* and *DNMTs* in leukemia patients, not only do these findings uncover an unconventional pathway for NCL oncogenic features, but also disclose a previously unidentified epigenetic regulator in leukemia cells, thus providing a profitable rationale for targeting NCL as epigenetic therapy. Notably, although our results demonstrated that NCL-dependent DNMT1 upregulation occurs through NFκB signaling, we cannot exclude these possibilities, (1) as a RNA stabilizer, NCL directly binds to and stabilizes DNMT1 mRNA to augment DNMT1 protein expression; (2) NCL directly transactivates *DNMT1* gene promoter, because NCL and Sp1 share very similar binding elements and NCL can independently bind to Sp1 binding sites [50], a transactivator enriched on *DNMT1* promoter [7]. The demonstration of such possibilities could greatly advance our understanding of NCL-modulated epigenetic aberrations in leukemogenesis. Thus, our findings highlight NCL as an innovative member of the epigenetic regulator family and offer a preclinical concept that targeting NCL-DNMT axis can significantly improve outcomes for leukemia patients with relapse or unresponsiveness to DNA hypomethylating agents.

## MATERIAL AND METHODS

### Plasmids, siRNAs and reagents

Human *NCL* expression plasmid (#28176) and *NFκBp65* expression plasmid (#21966) were purchased from Addgene. *DNMT1* gene promoter region containing NFκB binding site 5'-GGGGTATCCC-3' was cloned to pGL3 vector (pGL3-*DNMT1*) and the detailed information is provided in Supplementary Methods. On-target^plus^ smart pool siRNAs containing a mixture of 4 oligonucleotides with potential to target human *NCL*, *NFκBp65* or their scramble oligos were obtained from Dharmacon. AS1411 and its negative control CRO26 were synthesized by Integrated DNA Technologies. The sequences are listed in Supplementary Methods. Bay 11-7082 was purchased from Sigma Aldrich.

### Cell culture and transfection

Cell lines (293T, Kasumi-1 and MV4-11) were obtained from American Type Culture Collection (Manassas). Kasumi-1 and MV4-11 cell lines were cultured in RPMI 1640 and 293T cell line in DMEM supplemented with 20% (Kasumi-1) or 10% (others) fetal bovine serum (FBS) (Life Technology). Before transfection or treatment, Kasumi-1 and MV4-11 cells were cultured at a density of 0.3 × 10^6^/mL and seeded into 6-well plates overnight. The siRNA oligos were introduced into cells using Lipofectamine™ RNAiMAX reagent (Life Technologies) according to the manufacturer's instruction. Expression plasmids were introduced into Kasumi-1 and MV4-11 cells by electroporation using Gene Pulser Xcell electroporation system (Bio-Rad, Hercules, CA). 10 × 10^6^ of Kasumi-1 or MV4-11 cells were resuspended in 380-400 μL RPMI 1640 without FBS, mixed with 15-20 μg plasmid in a 4 mm electroporation cuvette, and then pulsed at 250 V, 950 μF for 28 ms.

### Clonogenic assays

The clonogenic assays were performed as previously described [1]. Briefly, at 6 hours after transfection or exposure to drugs, about 1,000 cells were subjected to methylcellulose colony formation assays in MethoCult^®^ mixture (Stem Cell Technologies Inc), following the manufacturer's instruction. The number of colonies, each consists of more than 50 cells, were scored in 7–14 days.

### Dotblot

The genomic DNA was purified using DNA blood/tissue Kit (Qiagen) and Dotblot was performed according to a previous report [49]. Briefly, ~ 2 μg of DNA was denatured in 1 × buffer (0.4 M NaOH, 10 mM EDTA) at 100 °C for 10 minutes and then neutralized with an equal volume of cold 2 M ammonium acetate (pH 7.0). The positive charged nylon membrane (Amersham) was pre-wet by placing the membrane gently at a 45 ° angle into a tray of 6 × saline sodium citrate (SSC) buffer and assembled onto the Bio-Dot apparatus (Bio-Rad) with a connection to the vacuum. After the membrane was rehydrated with H_2_O, the denatured DNA in a 50–200 μL solution was loaded onto the membrane. When the liquid has been filtered through, the membrane was washed with 2 × SSC buffer, air dried at room temperature, baked at 80 °C for 2 hours, and then blocked with 5% nonfat milk for 1 hour. The DNA spotted membrane was incubated with mouse anti-5mC antibody (Active Motif) at 4 °C overnight and the signal was detected by anti-mouse HRP-conjugated secondary antibody and enhanced chemiluminescence. The optical density was quantified with ImageJ Software. Of note, to determine whether the Dotblot has a quantitative feature, we purchased 5mC (100% pure) from ZYMO research (Irvine) and performed a series of dilution. The diluted 5mC was subjected to Dotblot following the protocol as described above.

### Western blot analysis

The Western blotting was carried out with whole cell lysates as previously described [1]. Briefly, upon different treatment, whole cell lysates were prepared by lysing the cells in 1× cell lysis buffer [20 mM HEPES (pH 7.6), 150 mM NaCl and 0.1% NP40] supplemented with 1 × Phosphatase Inhibitor Cocktail 2 and 3 (Sigma Aldrich), 1 mM PMSF (Sigma Aldrich) and 1 × protease inhibitors (protease inhibitor cocktail set III, Calbiochem-Novabiochem). The protein lysates were quantified, resolved by electrophoresis on sodium dodecyl sulfate (SDS)-polyacrylamide gel (4–12%, 7.5% or 7.5-15%), and transferred onto polyvinylidine difluoride membranes (Amersham). Equivalent gel loading was confirmed by probing with β-Actin antibody. The antibodies used were: -Actin and DNMT3A from Santa Cruz Biotechnology; phospho-NCL (Thr76/Thr84) from Biolengend; NCL, DNMT3B and NFκBp65 from Abcam; phospho-NFκBp65 (Ser536), cleaved Parp, cleaved caspase-3 and cleaved caspase-8 from Cell Signaling Technology; DNMT1 from New England Biolabs. The proteins recognized by the antibodies were visualized with an enhanced chemiluminescence Western blot detection system (Amersham).

### RNA isolation, cDNA preparation and quantitative PCR

The quantitative PCR was performed as previously described [1]. Briefly, according to the manufacturer's instructions, RNA was isolated using miRNeasy Kit (QIAGEN) and reverse transcription for cDNA was performed using high capacity cDNA reverse transcription Kit with RNase inhibitor (Life technologies). For the expression of *DNMT1, DNMT3A* and *DNMT3B*, quantitative real-time PCR was carried out by TaqMan^®^ gene expression assay (Applied Biosystems). The expression of *NCL* and *p15*^INK4B^ was measured by SYBR^®^ Green quantitative PCR. *18S* levels were analyzed as internal control for normalization. Expression of the target genes was measured using the ΔCT approach. The primers are listed in Supplementary Methods.

### Dual luciferase assay

*NFκB* or *NCL* expression vectors and pGL3-*DNMT1* plasmids were transiently cotransfected into 293T cells. As an internal control for normalization of transfection efficiency, a plasmid that expresses Renilla reinformis (SeaPansy) luciferase pRL-SV40, in which the luciferase gene is transcriptionally directed by simian virus 40 promoter, was cotransfected into cells. At 48 hours after transfection, dual luciferase assays were performed using Dual-Luciferase^®^ Reporter Assay System (Promega) according to the manufacturer's protocol. All values are ratios of luciferase/renilla and normalized to the activity of pGL3 empty vector.

### *In vivo* tumor growth

4–6 weeks old athymic nude mice (Harlan Laboratories) were used. All animal studies were performed in accordance with UMN institutional guidelines for animal care and under protocols approved by the UMN Institutional Animal Care and Use Committee. After *NCL* knockdown in MV4-11 for 6 hours, 2 × 10^6^ of transfected cells in 100 μL of 50% Matrigel^™^ (BD Biosciences) were injected subcutaneously into the flanks of nude mice. Vernier calipers were used to measure tumor size post-inoculation. The tumor diameters were measured at day 7 after injection and then every 2–3 days. End point tumor sizes were calculated by using the formula π/6 × A × B × C, where A is the length, B is the width, C is the height and expressed in cubic millimeter (mm^3^) [51]. The tumor tissues were fixed in 10% neutral buffered formalin for 24 hours at 4 °C and then transferred to phosphate buffered saline to make paraffin embedded blocks for H&E and IHC staining.

### Histopathological staining

Tumors collected from tumor-bearing nude mice were fixed in 10% neutral buffered paraformaldehyde, and then rinsed, dehydrated, embedded in paraffin and sectioned at a thickness of 5 μm. The tumor tissue slides were dewaxed, rehydrated and then routinely stained with Hematoxylin and Eosin (H&E) to examine general morphology or processed by IHC staining. The antibodies were: Ki-67, DNMT1, NCL, DNMT3A and DNMT3B from Abcam; phospho-NFκBp65 (Ser536) from Cell Signal; 5mC from Active Motif. The detailed description of IHC staining protocol is in Supplementary Methods.

### Bisulfite genomic sequencing

The genomic DNA was bisulfite-conversed by EpiTect Bisulfite Kit (Qiagen) according to the manufacturer's instruction. For subcloning and sequencing, bisulfite-treated genomic DNA was amplified. The primers are listed in Supplementary Methods. Purified PCR products were subcloned using the TA cloning Kit (Invitrogen). Individual clones were grown and DNA was prepared using QIAprep Spin Miniprep Kit. About 10 or 20 individual clones were subjected to sequencing with M13R primer in Genewiz. This assay generated a fragment of 251 bp of the *p15*^INK4B^ gene promoter encompassing 27 CpG sites located between nucleotide −4 and +247 relative to the transcription start. The *p15*^INK4B^ methylation frequencies of each sample were calculated as the number of methylated CpG sites divided by the total number of CpG sites sequenced per sample.

### Statistical analysis

All statistical analysis was carried out using GraphPad Prism 5.04. The reported numerical results represent the means ± SD (standard deviation) or ± SEM (standard error of mean). *P* value was determined by a two-tailed Student's t-test and differences were considered statistically significant at *P* < 0.05. The analysis of gene correlation was performed with Spearman correlation.

## SUPPLEMENTARY MATERIALS AND METHODS


