# Supplementary material for: A nucleolin-DNMT1 regulatory axis in acute myeloid leukemogenesis
Source: Oncotarget. 2014 Jun 26;5(14):5494–509. doi: 10.18632/oncotarget.2131 (PMC4170608; doi:10.18632/oncotarget.2131)
Supplement: Supplementary file 1 [file oncotarget-05-5494-s001.pdf]

A nucleolin-DNMT1 regulatory axis in acute myeloid leukemogenesis

Supplementary Materials and Methods

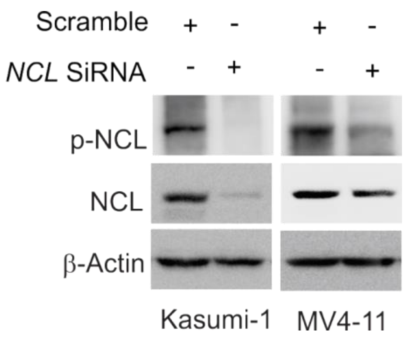

Supplementary Figure S1: *NCL* knockdown decreases its phosphorylation. MV4-11 or Kasumi-1 cells were transfected with *NCL* siRNA or scramble and the cells were lysed for Western blot 48 hours later. Note: p-NCL, phosphorylated NCL.

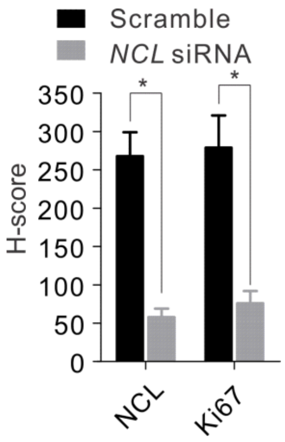

Supplementary Figure S2: Quantification of IHC staining reported in (Figure 2E) using H-score calculation. Bars represent mean + SD, *, $P < 0.05$.

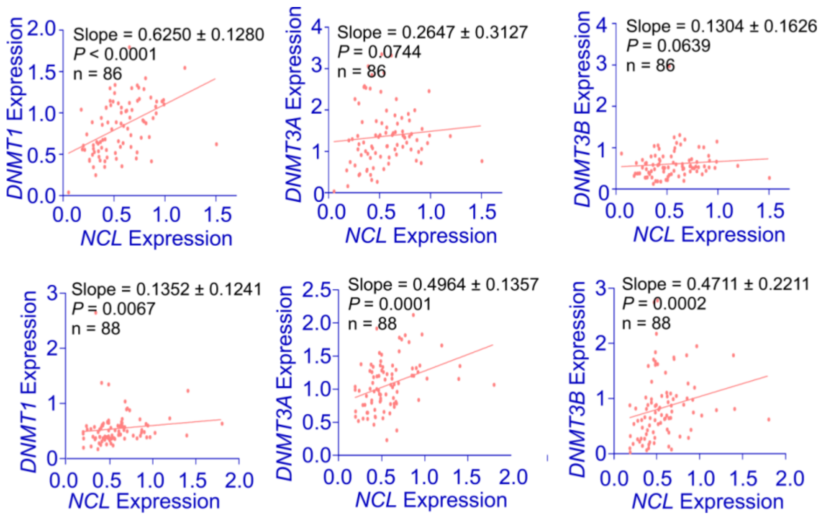

Supplementary Figure S3: The analysis of GEO datasets identifies the correlation of *NCL* and *DNMT* expression in leukemia patients. GEO datasets GSE16432 [GPL10105–10108, myeloid leukemia, n = 86 (upper); GPL8651–8653, myeloid leukemia, n = 88 (lower) [1]] were obtained and the correlation between *NCL* and *DNMT1* or *DNMT3A* or *DNMT3B* was assessed by the Spearman correlation. $P < 0.05$ was considered statistically significant.

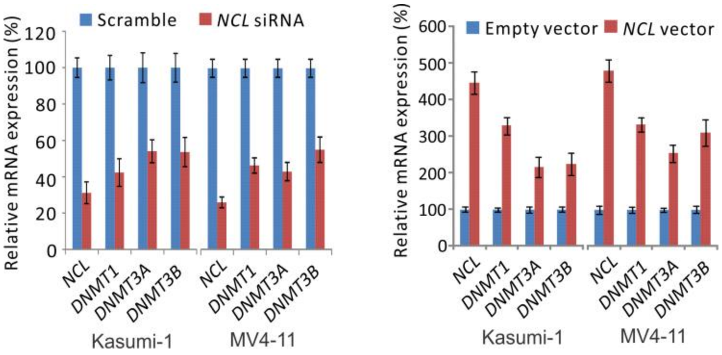

Supplementary Figure S4: NCL positively regulates the transcription of *DNMTs*. Left: MV4-11 or Kasumi-1 cells were transfected with *NCL* siRNA or scramble for 48 hours and the expression of targeted genes was detected by qPCR; Right: Kasumi-1 or MV4-11 cells were transfected with *NCL* expression or empty vector for 48 hours and the expression of targeted genes was detected by qPCR.

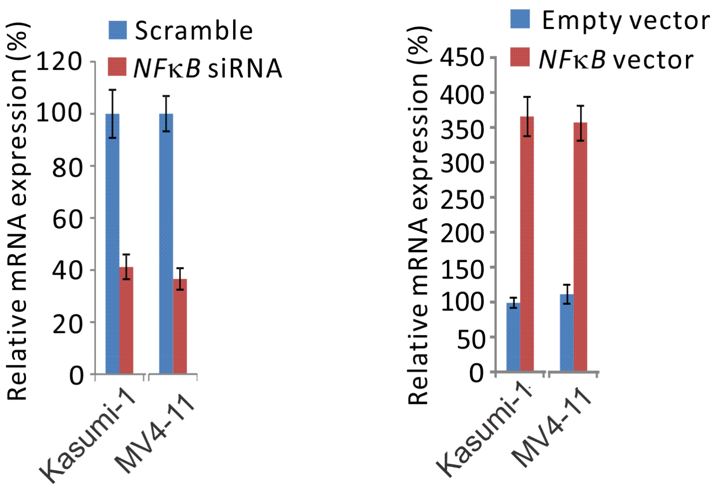

Supplementary Figure S5: NFκB positively regulates the transcription of *DNMT1*. Left: MV4-11 or Kasumi-1 cells were transfected with *NFκB* siRNA or scramble for 48 hours and the expression of *DNMT1* gene was detected by qPCR; Right: Kasumi-1 or MV4-11 cells were transfected with *NFκB* expression or empty vector for 48 hours and the expression of *DNMT1* gene was detected by qPCR.

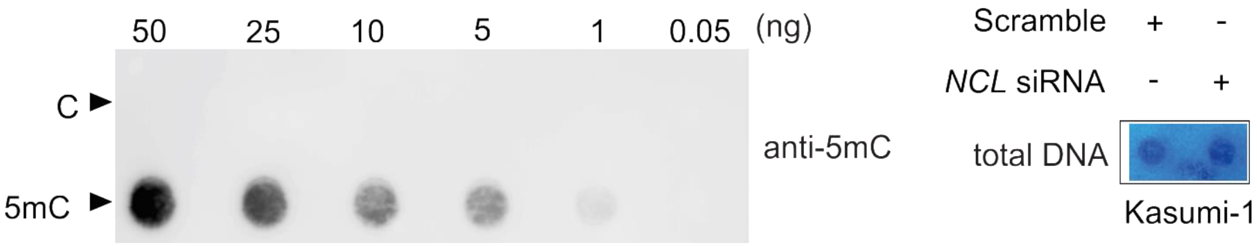

Supplementary Figure S6: The quantitative features of Dotblot. Left: Pure C and 5mC were diluted and spotted on nylon membrane, which was incubated with 5mC antibody. Right: Total DNA was spotted on nylon membrane and stained by methylene blue.

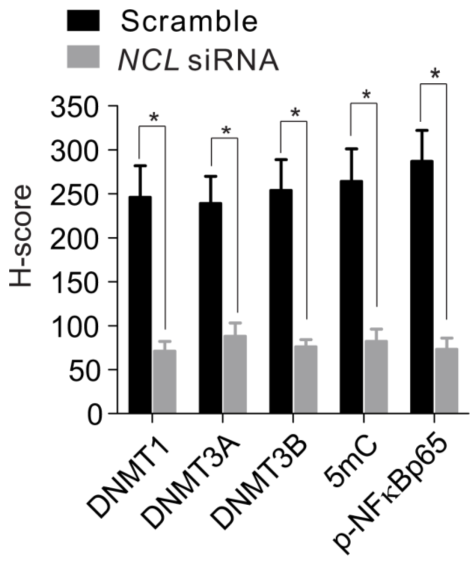

Supplementary Figure S7: Quantification of IHC staining reported in (Figure 5B) using H-score calculation. Bars represent mean + SD, *, $P < 0.05$.

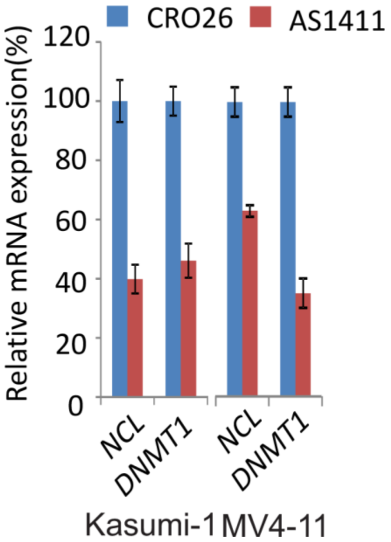

Supplementary Figure S8: AS1411 treatment decreases *DNMT1* expression. MV4-11 or Kasumi-1 cells were treated with AS1411 or its negative control CRO26 for 48 hours and the expression of *DNMT1* was detected by qPCR.

Plasmids, siRNAs and reagents

The human *DNMT1* gene promoter region (−1048/+36, relative to the transcription start +1), which contains NFκB binding site 5'-GGGGTATCCC-3' (-889/-880), was cloned to firefly luciferase reporter vector pGL3 (pGL3-*DNMT1*). Human genomic DNA from MV4-11 was used as template and the primers are:

forward 5'-GGGGTACCTACCCTGTCGCCCAGGCAGGAGTCCAGTGG-3';

reverse 5'-CCCAAGCTTGAGGCGATACCCTGTGCAGAAGGATGGAACG-3'.

The resulting PCR fragment (1048 bp) was subcloned in a KpnI/HindIII double-digested pGL3 basic vector (Promega Corp) and subjected to sequence analysis. The correct DNA fragment was subsequently cloned.

The sequences of NCL inhibitor are:

CRO26 5'-CCTCCTCCTCCTTCTCCTCCTCCTCC-3';

AS1411 5'-GGTGGTGGTGGTTGTGGTGGTGGTGG-3'.

RNA isolation, cDNA preparation and quantitative PCR

The primers used are:

NCL forward 5'-AAGGCACAGAACCGACTA-3';

reverse 5'-GACATCCACAACAGCAAGA-3';

p15^{INK4B} forward 5'-CCAGATGAGGACAATGAG-3';

reverse 5'-AGCAAGACAACCATAATCA-3';

18S forward 5'-ACAGGATTGACAGATTGA-3';

reverse 5'-TATCGGAATTAACCAGACA-3'.

Histopathological staining

For immunohistochemistry (IHC) staining, slides were deparaffinized through three baths of xylene for 5 min each before staining. Slides were rehydrated in a graded series of 100%, 90%, 70% and 50% (vol/vol) ethanol, and then finally hydrated in distilled water for 5 min each. All section slides were microwaved in

10 mM citric acid buffer (pH 6.0) at 70% power for 10 min to unmask antigens. Endogenous peroxidase was quenched using 3% hydrogen peroxide for 20 min followed by rinsing with PBS. Nonspecific binding was blocked with 10% goat serum for 40 min, then with avidin and biotin (Vector Laboratories) for 15 min each. Primary antibodies were incubated at 4 °C overnight. For detection of primary antibodies, the Vectastain Elite ABC-peroxidase Rabbit or Mouse IgG Kit (Vector Laboratories) was used. Samples were developed with 3, 3′-diaminobenzidine (Vector Laboratories), counterstained with hematoxylin, dehydrated and mounted in Vectamount (Vector Laboratories). Stained slides were viewed and photographed with a Leica microscope mounted with a high-resolution spot camera, which is interfaced with a computer loaded with Image-Pro Plus software. Sections known to stain positively were incubated in each batch and negative controls were also prepared by replacing the primary antibody with pre-immune sera.

The H-score is given as the sum of the percent staining multiplied by an ordinal value corresponding to the intensity level (0 = none, 1 = weak, 2 = moderate, and 3 = strong). With four intensity levels, the resulting score ranges from 0 (no staining in the tumor) to 300 (diffuse intense staining of the tumor). The difference was analyzed by GraphPad Prism 5.04, and $P < 0.05$ was considered significant.

Bisulfite genomic sequencing

The primers for $p15^{\mathrm{INK4B}}$ promoter are:

forward 5’-GGTTGGTTTTTTATTTTGTTAGAG -3’;

reverse 5’-ACCTAAACTCAACTTCATTACCCTC -3’.

References

1. Kharas MG, Lengner CJ, Al-Shahrour F, Bullinger L, Ball B, Zaidi S, Morgan K, Tam W, Paktinat M, Okabe R, Gozo M, Einhorn W, Lane SW, Scholl C, Frohling S, Fleming M, et al. Musashi-2 regulates normal hematopoiesis and promotes aggressive myeloid leukemia. Nature medicine. 2010; 16(8):903-908.
